# Phloxine B Staining is Compatible With High‐Throughput DNA Barcoding of Meiofauna

**DOI:** 10.1002/ece3.73597

**Published:** 2026-04-30

**Authors:** Romy Lisanne Gielings, Valentin Foulon, Willem Renema, Jan‐Niklas Macher

**Affiliations:** ^1^ Naturalis Biodiversity Center Leiden the Netherlands; ^2^ University of Amsterdam, Institute for Biodiversity and Ecosystem Dynamics (IBED) Amsterdam the Netherlands; ^3^ Univ. Brest, Ifremer, BEEP Plouzané France; ^4^ Leiden University, Institute of Environmental Sciences (CML), Department of Environmental Biology Leiden the Netherlands

**Keywords:** DNA barcoding, integrative taxonomy, meiobenthos, meiofauna, phloxine B, staining

## Abstract

Modern, integrative biodiversity research requires methods capable of bridging the gap between detailed morphological observations and the scalability of DNA sequencing. Integrative approaches like megabarcoding generate DNA sequences, while morphological functional data and abundance data are retained. For small, transparent, and highly abundant animals like meiofauna, the necessity to sort the specimens from organic matter and sediment forms a major bottleneck, as the use of stains required for enhanced manual or automated specimen detection can inhibit downstream molecular workflows. To address this, we tested the compatibility of phloxine B staining with DNA sequencing to facilitate simultaneous morphological and molecular specimen processing. Meiofauna preserved in ethanol, propylene glycol, or a DMSO/EDTA/saturated NaCl solution (DESS) was incubated with phloxine B for 30 min or 24 h. Specimens were manually isolated, the V1–V2 region of the 18S rDNA gene was sequenced, and success was quantified across major meiofaunal phyla. Nematodes, copepods, and annelids consistently showed high sequencing success irrespective of the preservative or staining incubation time. Platyhelminths showed lower success, likely due to misidentification or primer limitations rather than dye inhibition. Overall, these findings demonstrate that phloxine B is compatible with downstream DNA amplification and sequencing, enabling efficient integration of morphological and molecular data. The approach offers high potential for broader application in other microscopic taxa, supporting the way to comprehensive, high‐throughput biodiversity assessments or ecological monitoring.

## Graphical abstract

Modern, integrative biodiversity research requires methods capable of bridging the gap between detailed morphological observations and the scalability of DNA sequencing. For small, transparent, and highly abundant animals like meiofauna, sorting specimens from the matrix forms a major bottleneck, as the use of commonly used stains can inhibit downstream molecular workflows. Our findings demonstrate that phloxine B staining is compatible with DNA amplification and sequencing, enabling efficient integration of morphological and molecular data in meiofauna and supporting comprehensive, high‐throughput biodiversity assessments.

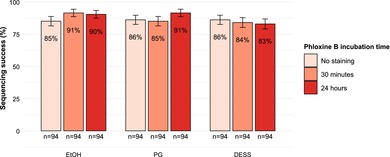

## 1 Introduction

Accurately and efficiently identifying and quantifying organisms across complex communities is a major challenge in biodiversity research. On the one hand, molecular techniques such as metabarcoding are powerful tools for large‐scale biodiversity assessments, but do not provide abundance data, and a lack of DNA references in public reference depositories impedes taxonomic assignment of sequences (de Faria et al. 2018; Fonseca et al. 2010; Lefaible et al. 2023; Macheriotou et al. 2021; Tang et al. 2012). On the other hand, morphological methods can provide information on functional traits, life stages, and absolute abundance, but are time‐consuming, labor‐intensive, and rely on taxonomic expertise, which often limits the temporal or spatial extent of these studies. Efficient integration of molecular and morphological methods is therefore essential, especially in systems composed of numerous small and morphologically similar organisms.

Recent developments such as megabarcoding (Chua et al. 2023) demonstrate the potential of this integration (Caterino and Recuero 2024; Hartop et al. 2024; Vihavainen et al. 2025). Originally introduced for insects, megabarcoding describes specimen‐based DNA barcoding of a bulk sample using a high‐throughput imaging and sequencing platform, generating DNA reference sequences, morphological and functional data, and abundance estimates simultaneously (Chua et al. 2023). By linking genetic and morphological information, megabarcoding not only strengthens ecological inference but also expands DNA reference libraries for metabarcoding applications.

Meiofauna are extremely abundant small (< 1 mm) animals that serve as key indicators of benthic ecosystem health (Alves et al. 2013; Semprucci, Frontalini, et al. 2015; Semprucci, Sbrocca, et al. 2015; Zeppilli et al. 2015). However, much of their diversity remains undescribed (Curini‐Galletti et al. 2012; Garraffoni et al. 2021). For both DNA barcoding and morphological identification, sorting meiofauna from sediment and organic matter is a time‐consuming process because of preservative‐related damage, their small size and often transparent bodies. For efficient sorting and taxonomic identification, staining of meiofauna is therefore a common practice (e.g., Ngo et al. 2013; Somerfield and Warwick 2013; Wang et al. 2019), as it greatly improves the distinction of the animals against the matrix (Mason and Yevich 1967). However, methodological incompatibilities between morphological and molecular workflows limit the integration of data, as common stains such as Rose Bengal inhibit DNA extraction and PCR success in nematodes regardless of the fixative used (Fonseca and Fehlauer‐Ale 2012), and contrasting results have been recorded for copepods (Easton and Thistle 2014; Watanabe et al. 2016). As these two taxonomic groups dominate meiofaunal communities, using staining in combination with molecular techniques is not recommended (Carugati et al. 2015; Fonseca and Fehlauer‐Ale 2012; van der Loos and Nijland 2021). This hinders the integration of morphological and molecular data, the broader application of molecular methods, and the upscaling of specimen processing pipelines by automated sorting.

Phloxine B, a xanthene dye related to fluorescein, may offer a promising solution. Phloxine B stains cytosolic material red to pink, has various applications in histology (Bencosme 1954; Lendrum 1947), and although less frequently than Rose Bengal, is used as a stain for benthic organisms (Bownes and Perissinotto 2012; Daché et al. 2024; Foulon et al. 2025; Mason and Yevich 1967; Shimabukuro et al. 2022). A stain compatible with integrated taxonomy approaches comprising molecular methods could provide a unified approach applicable to a wide range of ecological and taxonomic studies. Therefore, the goal of this study was to test the compatibility of phloxine B staining with DNA sequencing on individual meiofauna specimens. We assessed the effect of the preservatives ethanol (EtOH), propylene glycol (PG), and a solution of dimethyl sulfoxide (DMSO), EDTA, saturated with NaCl (DESS), and incubation times (30 min and 24 h) on the sequencing success across major meiofaunal phyla. We aimed to validate a specimen processing pipeline that facilitates efficient morphological sorting alongside high‐throughput DNA barcoding, thereby accelerating the upscaling of integrative biodiversity research.

## 2 Materials and Methods

One liter of sediment was collected on June 23, 2025, from a mussel bank located in the Dutch Wadden Sea island of Texel (53°09'N, 4°09'W), by scraping the first centimeter of sediment. The collected sediment was divided into three equal parts. To detach the organisms from the sediment, freshwater shock was induced for 2 min (Giere 2009), and the meiofaunal fraction was retained by decanting the suspension over a 1000‐μm and 41‐μm sieve cascade. The extracted meiofauna was stored in 30 mL of 70% PG, 96% EtOH or DESS (20% DMSO, 0.25 M EDTA, and saturated with NaCl; Yoder et al. 2006) at 4°C for 2 months before being processed for staining.

Phloxine B (RAL diagnostics) was added to two 5‐mL subsamples of each preservative to a final concentration of 1 mg/mL (0.1%), as this concentration was previously used for automated sorting (V. Foulon, personal communication). One subsample for each preservative was not stained and served as control. Tubes were wrapped in aluminum foil to prevent fading of the stain. One set of tubes was incubated in phloxine B for 30 min, and the other set for 24 h (Foulon et al. 2025).

After the respective incubation time, excessive staining was removed by washing the stained meiofauna over the 41‐μm sieve with their respective preservative, and the meiofauna was flushed into a petri dish. Specimens were sorted individually into PCR plates, containing 30 μL of 1X PCR buffer (Barlow 2019). Where possible, we filled six columns with nematodes (48), four columns with copepods (32), and two columns with other meiofaunal phyla (10) to approximate natural conditions where nematodes and copepods form the majority of the meiofaunal abundance in many habitats (e.g., Liu et al. 2025; Tachibana et al. 2023; Tong et al. 2022). Additionally, one negative extraction control containing only 30 μL of buffer and one well was left empty to serve as a negative template control during PCR. DNA extraction followed the protocol by Barlow (2019). In short, the PCR plates containing the specimens in 1X PCR buffer were placed at −80°C for 10 min and subsequently incubated in a thermocycler at 60°C for 70 min and at 95°C for 15 min. Plates were stored at −20°C until further processing. The V1–V2 region of the 18S rDNA gene was amplified using the SSU‐F04 (GCTTGTCTCAAAGATTAAGCC) and SSU‐R22 (GCCTGCTGCCTTCCTTGGA) primers (Blaxter et al. 1998). SSU‐F04 and SSU‐R22 are routinely used in metabarcoding studies for meiofauna as they allow for successful amplification of many meiofaunal taxa (Gielings et al. 2021).

The PCR was performed in a final volume of 20 μL containing 9.4 μL of nuclease‐free water (UltraPure), 4 μL of reaction buffer (Phire Green, 10×; Thermo Scientific), 0.8 μL of bovine serum albumin (Promega, 10 mg/mL), 0.4 μL of dNTPs (2.5 mM), 0.4 μL of DNA polymerase (Phire Green Hot Start II, Thermo Scientific), 1 μL of Oxford Nanopore‐tailed primers (10 pmol/μl) and 3 μL of template. Initial denaturation at 98°C for 30 s, 35 cycles of 98°C for 5 s, 57°C for 10 s and 72°C for 15 s and a final extension of 5 min at 72°C. Two rows of each PCR plate were checked for successful amplification on a 1% agarose gel. A bead cleanup was performed using the C. Wash (Cytena) using 0.9× magnetic bead volume (NucleoMag NGS Clean‐up and Size Select, Machery‐Nagel). An indexing PCR was performed using the Oxford Nanopore barcoding primers (BC01‐96) EXP‐PBC096 and in a 10 μL reaction volume containing 2.5 μL of the barcoding primers, 5 μL of LongAmp Taq 2× Master Mix (New England Biolabs), and 2.5 μL of the PCR template. Initial denaturation was 3 min at 95°C, 12 cycles of 95°C for 15 s, 62°C for 15 s and 65°C for 50 s, followed by final extension for 3 min at 65°C. For each plate, 1 μL per sample was pooled into a centrifuge tube. Bead cleanup was performed using magnetic beads at a 0.8:1 ratio. Pools were quantified using the 4150 Tapestation system with D5000 DNA ScreenTape (Agilent).

Library preparation was performed using the Oxford Nanopore Ligation Sequencing Kit V14 (SQK‐LSK114) and Native Barcoding Kit 24 V14 (SQK‐NBD114.24). The pool was sequenced on the Oxford Nanopore PromethION 2 Solo using R10.4.1 PromethION Flow Cells (FLO‐PRO114M). Super‐accurate basecalling was performed in MinKnow (v24.02.16) using Dorado (v1.3; Oxford Nanopore), and samples were demultiplexed using Guppy (v6.5.7), with “require barcode both ends” set to true. Bioinformatic processing was performed in Naturalis Galaxy platform (v25.0; The Galaxy Community 2024). Using a Snakemake pipeline, reads were filtered (minimum average read quality: 15, minimum size: 250, maximum size: 600); primers were identified and removed using Cutadapt with a max error rate of 20% and minimum overlap of 80%. Samples with fewer than 30 remaining reads were left out. Consensus sequences were generated using NGSpeciesID (v0.3.1; Sahlin et al. 2021) using the following settings: k—13, w—20, mapped threshold—0.7, aligned threshold—0.4, minimum fraction—0.8, min prob. no hits—0.1. Consensuses constructed with less than 5 reads or 1% of the sample's reads were discarded. The remaining consensus sequences were polished using medaka (v2.0.1); the medaka model was automatically detected from the FastQ description lines. Taxonomy was assigned to the reads against a subset NCBI Genbank version 258, by selecting only records containing the word “18S” in their description, and using a query coverage of at least 75% and identity percentage cutoff of 80%.

We defined a successful sequencing result as generation of a consensus sequence of more than 500 reads that constitute at least 80% of the total reads of that well, of which the read identification matches the expected meiofaunal phylum with at least 75% query coverage and 90% similarity to an existing reference sequence in NCBI Genbank. Before statistical tests, bivalves were removed from the dataset due to low sample size (*n* = 2). We then modeled the effect of preservative, incubation time, and taxonomic group on the number of successful sequencing results using generalized linear mixed models and the car::Anova() function. Plots were made using the ggplot2 package. All statistics were performed in Rstudio (version 4.5.1).

## 3 Results

Phloxine B staining produced bright pink individuals in both DESS and ethanol. In contrast, staining in PG was less consistent and of lower intensity. After 30 min of incubation, only a subset of individuals showed light staining. After 24 h, staining intensity increased; however, some specimens remained unstained, and some stained specimens remained noticeably lighter than specimens preserved in DESS or ethanol. Despite this, staining facilitated the handling of specimens, making it easier to locate the immobile specimens and verify their presence in the tube compared with the nonstained control samples.

**FIGURE 1:**
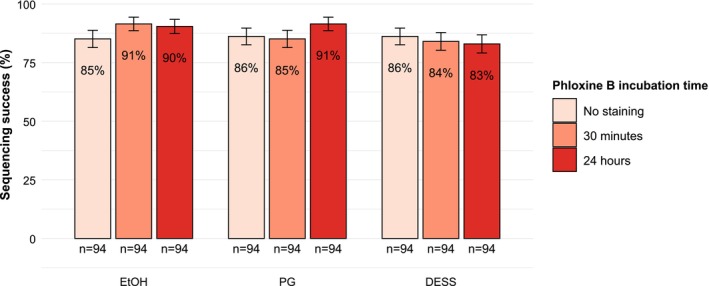
Sequencing success (+ − se) of specimens preserved in ethanol (EtOH), propylene glycol (PG), or DESS, and stained with phloxine B for different durations: no staining (control), 30 min, or 24 h.

A total of 846 specimens were sequenced, comprising nematodes (*n* = 495), copepods (*n* = 247), annelids (*n* = 79), platyhelminths (*n* = 23), and bivalves (*n* = 2). Overall sequencing success rates across different preservatives and incubation times ranged from 83% to 91% (Figure 1). The data revealed no significant interaction between preservative and staining duration (*p* = 0.77). Neither staining duration nor preservative significantly influenced sequencing success (*p* = 0.50 and *p* = 0.21, respectively).

**FIGURE 2:**
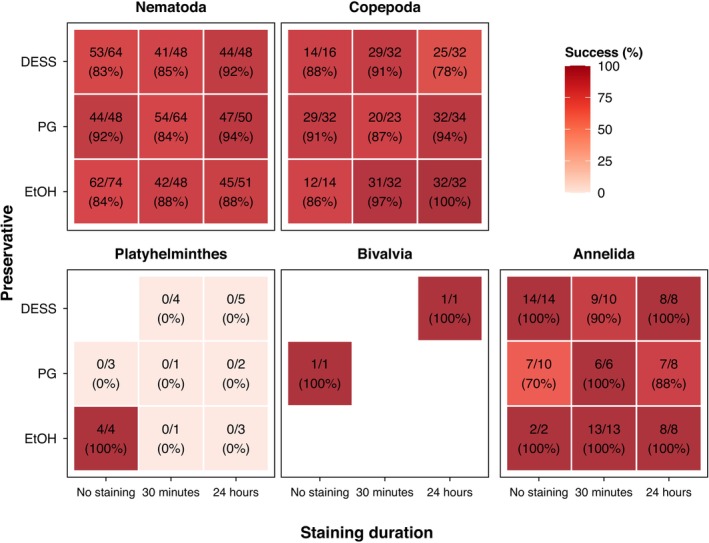
Sequencing success rates by taxon.

Taxonomy of the sequenced specimen had a strong effect on sequencing success (*p* < 0.001). Nematodes, copepods, and annelids showed high success rates across the different treatments (Figure 2). In particular, platyhelminths had significantly lower sequencing success than other taxa. Although for platyhelminths (*n* = 23 across all treatments) in 91% (*n* = 21) of samples a successful consensus was generated, the assigned taxonomy did not match the expected taxon, resulting in a final success rate of only 17% (*n* = 4). All successful sequences came from unstained ethanol‐preserved samples. Bivalve staining and sequencing were successful, but included only two individuals.

## 4 Discussion

Our study demonstrates successful DNA sequencing from individual specimens across major meiofaunal phyla that were subjected to staining with phloxine B, rendering the use of phloxine B compatible with molecular workflows and directly refuting the assumption that staining techniques are incompatible with DNA‐based analyses.

Sequencing success was independent of preservative and incubation time, but refining the method may be necessary. Firstly, while preservation in 96% EtOH yielded the only successful platyhelminth sequences, this EtOH concentration generally induces significant dehydration and morphological shrinkage (Marquina et al. 2021; Naem et al. 2010), compromising accurate taxonomic identification. We recommend that researchers select the preservative based on their primary research goals, such as maximizing morphological preservation or optimizing DNA integrity for long‐term storage. 70% or 80% ethanol may be potential alternatives to test for compatibility with phloxine B staining. Secondly, although no data was recorded, light and partial staining was observed across all phyla after incubation in PG. Possibly, the higher viscosity of PG delays the uptake of phloxine B in the tissue, requiring extended incubation times to achieve sufficient contrast for sorting. In contrast, phloxine B effectively stains organisms when added directly to EtOH‐preserved samples within 30 min with similar efficiency to formalin and aqueous solutions (V. Foulon, personal observation based on red fluorescence levels on the COPAS Vision Cytometer, Union Biometrica). However, phloxine B is not permanently fixed within the tissues. Stained specimens can undergo fading when transferred back into EtOH solutions without stain, possibly resulting from leaching of phloxine B into the preservative. The rate and intensity of fading are sample‐dependent.

Taxonomy was the most important predictor for sequencing success. While nematodes, annelids, and copepods showed high, consistent success rates, platyhelminths showed significantly lower success. The substantial discordance between the expected and realized taxonomic identity of those individuals suggests either misidentification during the sorting or off‐target amplification. Freshwater shock, selected to extract adhesive intertidal taxa, likely induced osmotic shrinkage leading to misidentification during sorting. Alternatively, the potential low efficiency of the SSU4/R22 primers in platyhelminths could result in amplification of nontarget DNA. Gut contents of platyhelminths, functioning as predators and scavengers (Armonies 1986; Menn and Armonies 1999; Noreña et al. 2015), may potentially outcompete host DNA, leading to a false taxonomic assignment. Although neither explanation supports active inhibition by phloxine B, this potential effect should be explored further with more specific platyhelminth primers. In addition, gentler extraction methods such as magnesium chloride relaxation, thereby minimizing lysis and shrinkage, could be used in future studies targeting highly sensitive soft‐bodied taxa to evaluate compatibility with phloxine B staining.

## 5 Conclusion

We show that phloxine B staining directly supports the upscaling of meiofauna ecological and biodiversity studies by enabling simultaneous, high‐throughput morphological identification and molecular barcoding, paving the way for faster, standardized, and more comprehensive biodiversity assessment across large sample numbers. Beyond marine benthic meiofauna, this approach holds strong potential for other microscopic taxa in which sorting remains a bottleneck, including soil (micro)fauna and foraminifera. Applying this workflow in these systems could similarly accelerate taxonomic resolution, reduce processing time, and broaden the scope of integrative biodiversity monitoring across a wide range of environments.

## Author Contributions

**Romy Lisanne Gielings:** conceptualization (equal), data curation (lead), formal analysis (lead), investigation (lead), methodology (equal), visualization (lead), writing – original draft (lead). **Valentin Foulon:** conceptualization (equal), methodology (equal), supervision (equal), writing – original draft (supporting), writing – review and editing (equal). **Willem Renema:** conceptualization (equal), methodology (equal), supervision (equal), writing – original draft (supporting), writing – review and editing (equal). **Jan‐Niklas Macher:** conceptualization (equal), data curation (supporting), formal analysis (supporting), methodology (equal), supervision (equal), writing – original draft (supporting), writing – review and editing (equal).

## Funding

This work was supported by Sasakawa Peace Foundation.

## Disclosure

Sample collection and analyses were conducted in Europe, where all authors are based. The authors recognize the importance of equitable research partnerships and remain committed to fostering inclusive approaches in future projects.

## Conflicts of Interest

The authors declare no conflicts of interest.

## Data Availability

All data and code have been uploaded to figshare (doi:10.6084/m9.figshare.31047427). Barcode sequences have also been uploaded to NCBI GenBank, accession numbers PX722042—PX722877.
